# Case Report: Scar‐Like Alterations of the Gastric Mucosa Observed Under Endoscopy in Collagenous Gastritis

**DOI:** 10.1002/ccr3.72393

**Published:** 2026-04-09

**Authors:** Qian Luo, Yujie Li, Jiemin Liu, Xiaoyuan Lin, Sainan Zhou

**Affiliations:** ^1^ Department of Spleen and Stomach Diseases The First Hospital of Hunan University of Chinese Medicine Changsha China; ^2^ Department of Geriatrics, Inner Mongolia Inner Mongolia Hospital of Traditional Chinese Medicine Hohhot China; ^3^ Department of Digestive Endoscopy Guizhou Provincial People's Hospital Guiyang China; ^4^ Center for Medical Research and Innovation The First Hospital of Hunan University of Chinese Medicine Changsha China

**Keywords:** case report, collagenous gastritis, Masson's trichrome staining, scar‐like alterations, traditional Chinese medicine therapy

## Abstract

Collagenous gastritis (CG) is an uncommon disorder of the digestive system, characterized by marked thickening of the subepithelial collagen band and infiltration of inflammatory cells within the lamina propria. The etiology and pathogenesis of CG remain not fully understood, and its diagnosis is contingent upon histopathological evaluation, contributing to its status as an under‐recognized condition. This article presents a case involving a 35‐year‐old male patient of Chinese descent, whose primary clinical manifestations included upper abdominal bloating and discomfort. Endoscopic assessment revealed diffuse scar‐like alterations throughout the cardia, fundus, corpus, antrum, pylorus, and duodenal bulb. Histopathological analysis using Hematoxylin and Eosin (HE) staining indicated extensive inflammatory cell infiltration in the lamina propria along with focal fibrosis. Masson trichrome staining identified a subepithelial collagen fiber band with a maximum thickness of 34 μm, thereby confirming the diagnosis of CG. The patient's clinical symptoms showed significant improvement following treatment with a modified traditional Chinese herbal decoction, Banxia Xiexin Tang (BXXXT). This case reveals the heterogeneity of endoscopic manifestations in CG, emphasizes the importance of diffuse scar‐like changes as a potential endoscopic feature, and highlights the significant value of periodic endoscopic follow‐up for monitoring disease progression.

## Key Clinical Message

This study documents an uncommon endoscopic presentation of scar‐like changes in the gastric mucosa of a patient diagnosed with CG, highlighting the critical importance of endoscopy in the early diagnosis of CG and underscoring the necessity of regular endoscopic surveillance to monitor disease progression.

## 1 Introduction

Collagenous gastritis (CG) is an exceedingly rare inflammatory disorder characterized by the deposition of collagen fibers within the subepithelial layer of the gastric mucosa, accompanied by inflammatory cell infiltration in the lamina propria [1]. Epidemiological data reveal a remarkably low incidence, with only 13 cases of CG identified per 100,000 endoscopic examinations [2]. The etiology of CG remains unclear, though it is likely multifactorial, involving potential associations with immune regulation imbalances, inflammatory stimuli, pharmaceutical effects, and genetic predispositions [3]. In clinical settings, the manifestations of CG are nonspecific, rendering diagnosis heavily reliant on histopathological examination. Clinically, Lagorce‐Pages et al. have categorized CG into two phenotypes: a pediatric/adolescent type, which presents with anemia and abdominal pain and is often confined to the stomach with nodular mucosa observed on endoscopy; and an adult type, which presents with diarrhea and weight loss and is frequently associated with collagenous colitis and other autoimmune diseases [4]. However, with the accumulation of cases, atypical presentations are increasingly reported. While the classic endoscopic appearance of CG is mucosal nodularity, findings such as erythema, erosions, atrophy, and even normal appearances have been documented [5, 6, 7].

Due to the rarity and clinical heterogeneity of CG, the current understanding of this condition is predominantly based on case reports and retrospective analyses. There is a pressing need to gather additional case data to improve medical recognition of CG. This article presents a case study of an adult Chinese male with CG, characterized by diffuse endoscopic scar‐like changes and rapid disease progression. The aim is to enhance clinicians' comprehension of CG endoscopic phenotypes and to decrease the incidence of missed diagnoses and misdiagnoses.

## 2 Case Description

A 35‐year‐old male of Chinese descent presented with symptoms of upper abdominal bloating and discomfort, accompanied by intermittent episodes of acid reflux, heartburn, and postprandial nausea and vomiting. Physical examination did not reveal any significant abnormalities. The patient had no notable past medical history and reported no history of long‐term medication use.

**FIGURE 1:**
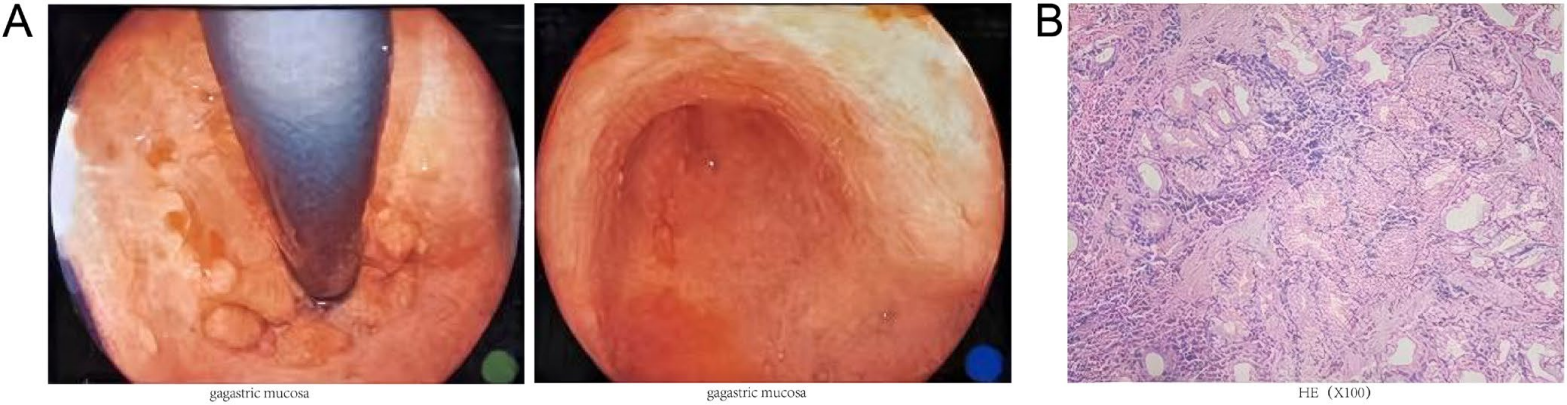
Initial Endoscopic and Pathological Assessment (January 16, 2025). (A) Loss of mucosal folds throughout the stomach accompanied by rigid peristalsis. (B) Pathological findings: Small areas of active glandular epithelial hyperplasia and interstitial infiltration with a significant presence of inflammatory cells, predominantly lymphocytes, plasma cells, neutrophils, and eosinophils.

The patient first initially sought medical attention at a local hospital on January 16, 2025, for symptoms of “upper abdominal bloating.” Laboratory investigations, including a complete blood count and assessments of liver and kidney function, were unremarkable. A *Helicobacter pylori* breath test returned negative results. Serum pepsinogen analysis indicated a decreased Pepsinogen I (PG I) level of 22.37 ng/mL, a Pepsinogen II (PG II) level of 6.22 ng/mL, and a reduced PG I/PG II ratio of 3.60. Ultrasound imaging of the liver, gallbladder, spleen, and pancreas did not reveal any abnormal acoustic findings. The initial endoscopic examination suggested the disappearance of mucosal folds throughout the stomach and rigid peristalsis. The differential diagnosis considered by the physician included “autoimmune gastritis” and “mucosa‐associated lymphoid tissue (MALT) lymphoma” (Figure 1A). The pathology report indicated “chronic superficial gastritis” without the presence of tumor cells or characteristic atrophy, which was inconsistent with the severe findings observed during endoscopy (Figure 1B). Given the ambiguous diagnosis and the limitations of the local medical facilities, the physician advised transferring the patient to a higher‐level hospital for further evaluation.

**FIGURE 2:**
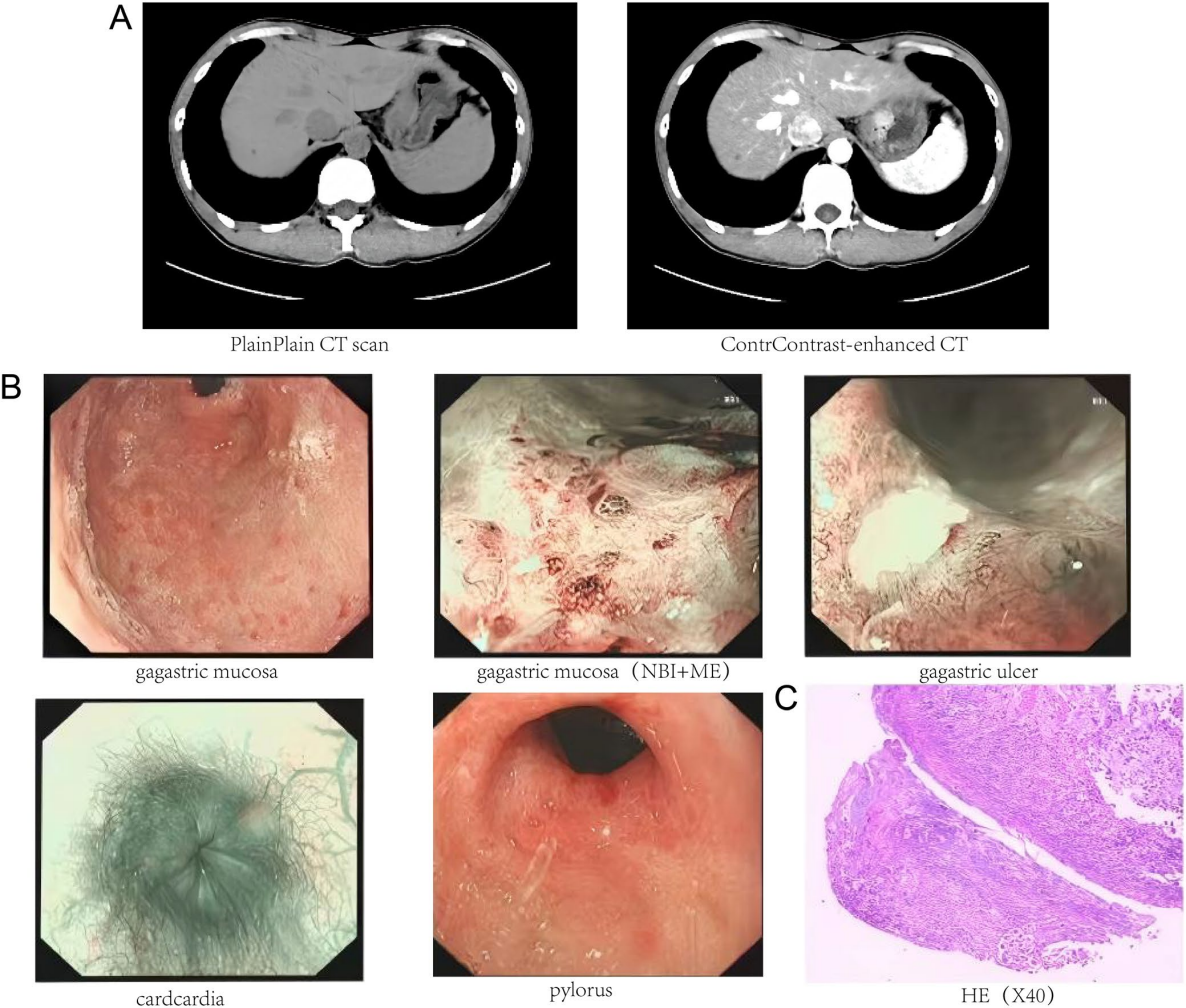
Abdominal Contrast‐Enhanced CT, Second Endoscopic and Pathological Examination (January 22, 2025). (A) There is pronounced thickening of the gastric wall near the cardia. The enhanced scan reveals multiple small lymph nodes within the perigastric region. (B) The gastric mucosa exhibits total atrophy, with multiple scar‐like alterations. A substantial depressed ulcer is observed on the anterior wall at the junction of the antrum and corpus. (C) Pathological examination reveals ulcer formation with atypical cells present within necrotic tissue, alongside mild atypical hyperplasia in certain glands.

On January 21, 2025, the patient sought medical attention at an external hospital. Subsequent follow‐up serum gastric function tests revealed a PG I level of 8.41 ng/mL, which is decreased, a PG II level of 6.30 ng/mL, and a PG I/PG II ratio of 1.33, also decreased, suggesting a deterioration in gastric mucosal atrophy. A contrast‐enhanced computed tomography (CT) scan of the abdomen demonstrated marked thickening of the gastric wall near the cardia at the fundus, characterized by irregular margins, slight narrowing of the gastric lumen, and rigidity of the mucosal surface. Enhanced imaging showed significant enhancement of the gastric wall near the cardia, with a relatively smooth serosal surface, and the presence of multiple small lymph nodes in the perigastric region surrounding the fundus (Figure 2A). A subsequent endoscopic examination revealed complete gastric mucosal atrophy characterized by a rough, uneven surface and multiple scar‐like alterations. A depressed ulcer, approximately 0.6 cm in diameter, was identified on the anterior wall at the junction of the antrum and corpus. Under Narrow Band Imaging (NBI) combined with Magnifying Endoscopy (ME), the microvessels appeared dense, tortuous, and irregular; however, the demarcation line, microvascular pattern, and mucosal surface pattern were all negative. Additionally, the antral cavity was reduced, the cardia mucosa exhibited noticeable whitening, and the pylorus was slightly deformed (Figure 2B). Notably, the gastric mucosa was firm in texture and susceptible to spontaneous bleeding upon inflation. Histopathological analysis indicated ulcer formation and necrosis, while immunohistochemistry excluded lymphoma (CD3‐, CD20+, Ki67+, EBER‐), revealing only atypical hyperplasia (Figure 2C). Based on these findings, the physician hypothesized that the patient's bloating was attributable to a “gastric ulcer” and prescribed anti‐ulcer therapy with Rabeprazole Enteric‐coated Tablets, 20 mg once daily for six weeks. However, following the treatment period, the patient's bloating did not demonstrate significant improvement.

**FIGURE 3:**
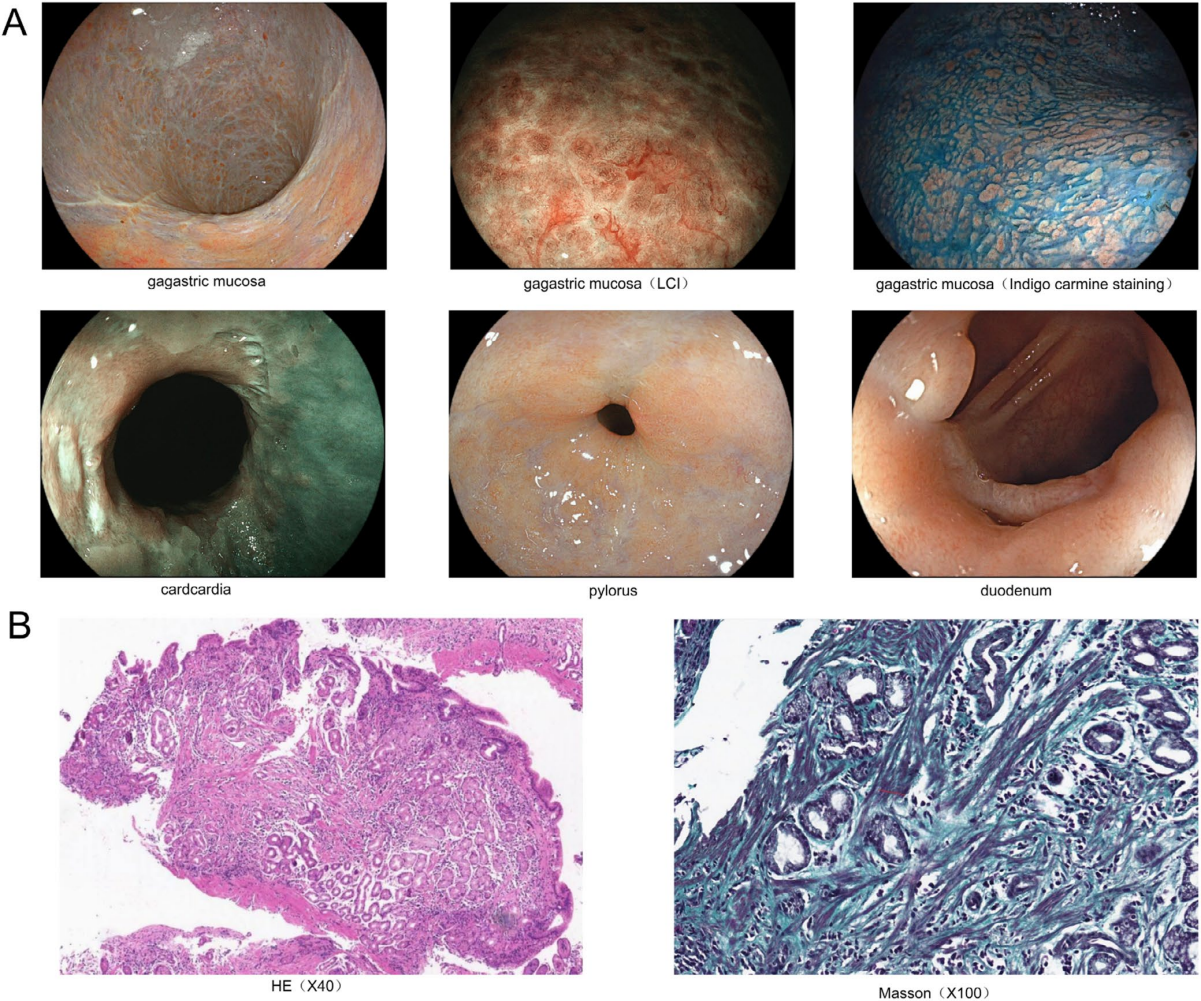
Illustrates the findings from the third endoscopic and pathological examination conducted on April 16, 2025. (A) Depicts diffuse gastric lesions exhibiting scar‐like alterations, stenosis of the cardia and pylorus with similar changes, and deformation of the duodenal bulb characterized by diverticula and bridging mucosa formation. Panel G presents HE staining, highlighting inflammatory cell infiltration and fibrous tissue proliferation within the lamina propria. (B) Displays Masson staining, revealing multiple collagen fiber bands, with the thickest band measuring approximately 34 μm.

**FIGURE 4:**
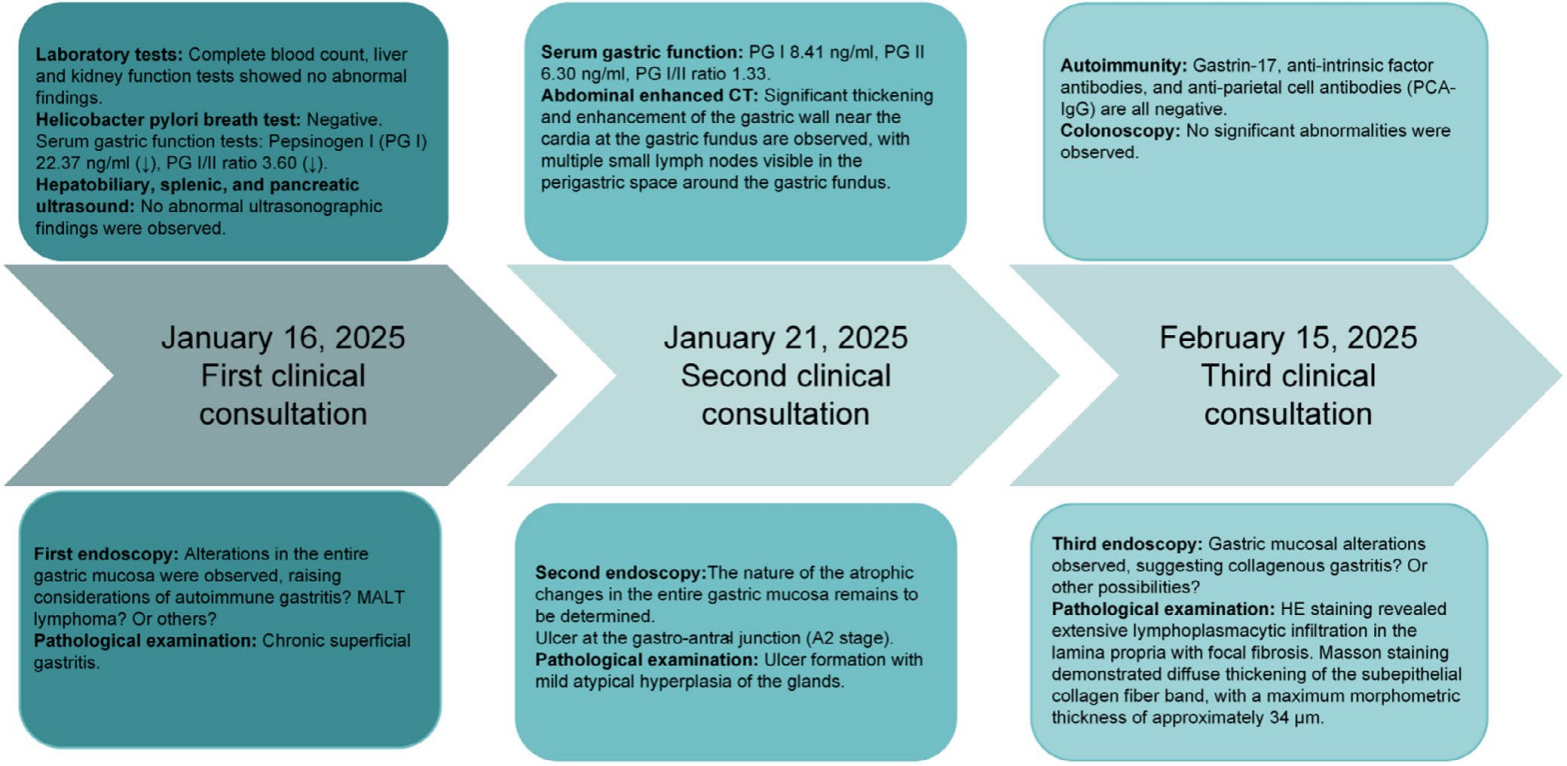
Patient Timeline.

The patient was admitted to our hospital on February 5, 2025. Comprehensive autoimmune screening was conducted, revealing normal levels of Gastrin‐17, anti‐intrinsic factor antibodies, and anti‐parietal cell IgG antibodies, thereby excluding the diagnosis of autoimmune gastritis. A third endoscopic examination identified diffuse lesions throughout the stomach, characterized by extensive scar‐like changes across all four walls, extending from the fundus to the corpus. Under Linked Color Imaging (LCI), the mucosa exhibited a whitish, atrophic appearance, with the residual mucosa displaying a “dried‐up” appearance, slight surface hyperemia, and edema, although the Regular Arrangement of Collecting Venules (RAC) remained distinct. Additionally, stenosis was noted in the cardia and pylorus, accompanied by scar‐like alterations; the duodenal bulb was markedly deformed, with diverticula and bridging folds present on the greater curvature and anterior wall. Colonoscopy revealed no significant abnormalities, thereby excluding collagenous colitis. To confirm the diagnosis, deep biopsies were obtained from the areas exhibiting the most pronounced scar‐like lesions (Figure 3A). Hematoxylin and Eosin (HE) staining revealed significant infiltration of lymphocytes and plasma cells, accompanied by a smaller presence of neutrophils and eosinophils within the lamina propria, alongside localized fibrous tissue proliferation. Masson staining indicated a diffuse thickening of the subepithelial collagen fiber band, with a maximum thickness measured at approximately 34 μm, consistent with the diagnostic criteria for CG (Figure 3B). Timeline chart of the patient's diagnosis and treatment (Figure 4).

## 3 Therapeutic Intervention

In light of the patient's non‐responsiveness to proton pump inhibitors (PPIs) and the current lack of standardized therapeutic guidelines for CG, a treatment strategy based on Traditional Chinese Medicine (TCM) was implemented. According to TCM principles, the patient's pathogenesis was diagnosed as “intermingled cold and heat, and dysfunction of ascending and descending Qi,” aligning with the Banxia Xiexin Tang syndrome. Consequently, a modified decoction of Banxia Xiexin Tang (BXXXT), comprising Pinellia, Scutellaria, Coptis, Dried Ginger, Codonopsis, Roasted Licorice, and Jujube, was prescribed.

During the initial consultation on February 5, 2025, the patient was administered a modified version of BXXXT, which included Processed Ginger, Codonopsis, Jujube, Bran‐fried Immature Bitter Orange, Tangerine Peel, Coptis, Pinellia, Scutellaria, and Roasted Licorice. At the subsequent follow‐up on February 17, 2025, the patient exhibited a significant reduction in bloating compared to baseline measurements, and the TCM regimen was adjusted to include Codonopsis, Jujube, Ginger Processed Magnolia Officinalis, Tangerine Peel, Pinellia, Scutellaria, Bran‐fried Atractylodes, Roasted Licorice, and Processed Ginger. By the third visit on April 16, 2025, the patient reported an absence of specific discomfort, prompting the continuation of TCM therapy to consolidate the therapeutic effects with the same formulation, supplemented by Nardostachys. At the follow‐up on June 17, 2025, the patient continued to experience no specific discomfort.

## 4 Discussion

CG is an uncommon disorder characterized by the deposition of collagen within the gastric tissue. The etiology and pathogenesis of CG remain incompletely understood, and its diagnosis is primarily dependent on histopathological evaluation; thus, it continues to be a poorly comprehended condition [3]. The clinical presentation of CG is varied and nonspecific. In addition to prevalent symptoms such as anemia, abdominal pain, diarrhea, and weight loss, less frequent symptoms such as bloating, dyspepsia, nausea, vomiting, fatigue, retrosternal pain, dysphagia, constipation, edema, gastrointestinal bleeding, and perforation have been documented. Some patients may even be asymptomatic, which complicates the clinical diagnosis significantly [8, 9].

The definitive diagnosis of CG is based on histopathological findings, specifically the presence of a subepithelial collagen band exceeding 10 μm in thickness, along with inflammatory cell infiltration in the lamina propria [10]. Endoscopically, a nodular appearance of the gastric mucosa is a relatively specific feature of CG, observed in approximately 60.2% of patients, while about 26.1% exhibit erythema or erosions. Less common manifestations include atrophy, ulcers, and hemorrhage [11]. Moreover, rare manifestations such as a cobblestone pattern or pseudo‐polyp‐like changes have been documented [12, 13]. The most notable feature of this case was the diffuse “scar‐like change” observed during endoscopy, characterized by linear or reticular white scars reminiscent of severe atrophic gastritis. However, pathological analysis did not reveal evidence of glandular atrophy, and the RAC was clear. This indicates that “scar‐like change” may represent a distinct and frequently overlooked endoscopic feature of CG. These scar‐like changes offer a novel diagnostic approach for CG, which may be clinically mistaken for the late‐stage atrophy associated with autoimmune gastritis. Although the markedly reduced PG I levels in this patient suggested atrophy, the absence of autoantibodies and the lack of characteristic parietal cell loss in pathology, along with positive Masson staining, were crucial in differentiating between the two conditions. Importantly, CG patients are susceptible to spontaneous mucosal bleeding during inflation in gastroscopy, indicating that gastric distension may serve as a mechanical injury factor potentially leading to gastrointestinal hemorrhage. To prevent iatrogenic bleeding, it is essential to maintain moderate inflation during endoscopy in patients suspected of having CG. Furthermore, the progression of the disease was distinctly observable across the three endoscopic examinations conducted in this case, highlighting the potential for rapid advancement of mucosal lesions in CG. Regular endoscopic evaluations are therefore crucial for monitoring changes in the disease.

The pathogenesis of CG remains poorly understood. Clinical observations have frequently noted comorbidity between CG and autoimmune diseases such as systemic lupus erythematosus, celiac disease, microscopic colitis, and autoimmune thyroid disease [14]. Moreover, gene expression profiling studies have revealed that Th1 and Th2 cytokine‐related genes are simultaneously upregulated in the gastric tissues of CG patients, distinguishing it from other forms of chronic gastritis and collagenous colitis. This suggests that immune factors may play a significant role in the pathogenesis of CG [15]. The inflammatory response constitutes a fundamental mechanism in this context. The infiltration of lymphocytes, plasma cells, and eosinophils within the lamina propria, coupled with the predominance of type III collagen in the deposited collagen band—indicative of repair processes—suggests an evolutionary progression characterized by “inflammation‐protein leakage‐fibrosis” [6]. Inflammatory mediators have the capacity to activate endothelial cells, thereby enhancing microvascular permeability and facilitating the leakage of plasma proteins such as fibrinogen and albumin into the submucosa. Simultaneously, these leaked protein components serve as chemotactic agents, recruiting additional inflammatory cells and activating local immune responses, which compromise the integrity of the mucosal barrier and stimulate the differentiation of fibroblasts into myofibroblasts. Myofibroblasts subsequently secrete type I and type III collagen; however, an imbalance between matrix MMPs and tissue inhibitors of TIMPs results in diminished collagen degradation and increased deposition, culminating in the formation of a dense, acellular collagen band [16, 17]. Furthermore, factors such as diet, medication, environmental influences, and genetic predispositions play significant roles in the pathogenesis of this condition [18].

Due to the infrequency of CG, treatment strategies are predominantly informed by case reports and empirical evidence, with a primary focus on alleviating symptoms and enhancing quality of life. Pharmacological interventions typically include PPIs, H2 receptor antagonists, iron supplements, immunosuppressants, and corticosteroids [19]. Nevertheless, considering the chronic and recurrent characteristics of CG, the potential for drug‐related adverse effects necessitates careful consideration during prolonged medication use. Non‐pharmacological approaches encompass dietary modifications and total parenteral nutrition [20]. In the present case, the patient initially underwent proton pump inhibitor therapy for ulcer management; however, there was no significant improvement in bloating. Consequently, treatment was shifted to TCM utilizing the BXXXT formula, with modifications tailored to the patient's symptoms. BXXXT, a classical formulation, is reputed for its ability to “harmonize the liver and spleen, balance cold and heat, and disperse accumulation to relieve distention.” It is frequently employed in clinical settings for managing digestive system disorders such as chronic gastritis, functional dyspepsia, peptic ulcers, and *Helicobacter pylori* infection [17, 18, 19]. Recent pharmacological research suggests that BXXXT can suppress the expression of inflammatory mediators such as TNF‐α, IL‐2, and IL‐8, and modulate the Th17/Treg balance, thereby mitigating mucosal inflammation [21]. Additionally, components of BXXXT, specifically Licorice (Glycyrrhiza) and Scutellaria (Scutellaria baicalensis), have demonstrated anti‐fibrotic properties, potentially reducing collagen deposition through the inhibition of the TGF‐β1/Smad signaling pathway [22, 23]. This case underscores the potential of alternative medicine as a novel approach for the treatment of CG.

## 5 Conclusion

This case report broadens the phenotypic spectrum of collagenous gastritis, establishing that scar‐like changes represent a rare yet noteworthy endoscopic manifestation. Clinicians should maintain a heightened level of suspicion for CG in patients exhibiting atypical atrophy, stiffness, or unexplained scarring during endoscopy, even in the absence of characteristic nodular alterations. Routine application of Masson trichrome staining is recommended for definitive diagnosis. Additionally, regular endoscopic surveillance is essential for monitoring the progression of this rapidly advancing fibrotic condition.

## Author Contributions

**Luo Qian:** conceptualization. **Yujie Li:** writing – review and editing. **Jiemin Liu:** writing – review and editing. **Xiaoyuan Lin:** conceptualization, writing – review and editing. **Sainan Zhou:** conceptualization, writing – review and editing.

## Funding

National Natural Science Foundation of China (No. 82060850); Natural Science Foundation of Hunan Province (No. 2024JJ9435); Joint Fund Project of the Natural Science Foundation of Hunan Province for the Medical and Health Industry (No. 2025JJ80976); Natural Science Foundation of Inner Mongolia Autonomous Region (No. 2024QN08057); Traditional Chinese Medicine (TCM) Scientific Research Plan Fund Project of Hunan Province (No. B2024047); Changsha Municipal Natural Science Foundation (No. kq2403098); Open Fund of the Cultivation Base for the State Key Laboratory, by the Province and Ministry for TCM Powder and Innovative Drugs (No. 23PTKF1010); University‐level Graduate Innovation Research Project of Hunan University of Chinese Medicine (No. 2024CX209); Key Specialty of Traditional Chinese Medicine (Category I) in Hunan Province; First‐class Domestic Construction Discipline of Traditional Chinese Medicine at Hunan University of Chinese Medicine.

## Consent

Informed written consent was obtained from the patient for publication of this report and any accompanying images.

## Conflicts of Interest

The authors declare no conflicts of interest.

## Data Availability

Data sharing not applicable to this article as no datasets were generated or analysed during the current study.
